# Ovotransferrin as a Multifunctional Bioactive Protein: Unlocking Its Potential in Animal Health and Wellness

**DOI:** 10.3390/vetsci12060514

**Published:** 2025-05-24

**Authors:** Sahdeo Prasad, Bhaumik Patel, Prafulla Kumar, Jeffrey Kaufman, Rajiv Lall

**Affiliations:** 1 RD Life Sciences LLC, 8801 Enterprise Blvd, Largo, FL 33773, USA; 2 Department of Immunotherapeutics and Biotechnology, Texas Tech University Health Science Center, Abilene, TX 79601, USA

**Keywords:** ovotransferrin (OVT), antimicrobial, antioxidant, immunomodulatory, livestock animals

## Abstract

Ovotransferrin (OVT) is one of the major proteins of egg white and is known to bind and transport irons in animals. OVT exerts bacteriostatic and bactericidal activities due to its iron-binding capacity. OVT effectively controls the growth of various pathogenic microorganisms, including Pseudomonas, *E. coli*, Staphylococcus, Proteus, and Klebsiella species, as well as inhibiting the replication of viruses. OVT also has antioxidant, anti-inflammatory, anti-hypertensive, anticancer, and immuno-stimulating properties. For instances, OVT quenches free radicals, induces antioxidant enzymes, suppresses pro-inflammatory cytokines, increases immune cells, reduces angiotensin-converting enzymes, and inhibits the proliferation of cancer cells. In this review, the beneficial effects of OVT in both in vitro and in vivo, particularly livestock, are described. Because of its antimicrobial properties, OVT supplementation in livestock feed would be an excellent alternative to antibiotics, which reducing the development of antibiotic-resistant pathogenic microorganisms. Thus, OVT could be a game-changing protein for the growth, performance, and healthy life of animals.

## Simple Summary

Ovotransferrin (OVT) is a protein found in egg white and has a wide range of functional properties. It holds promise as an animal feed additive because it kills pathogenic bacteria. OVT binds to iron, which cause the starving of bacteria and the inhibition of their growth. As a natural antimicrobial agent, OVT can be used to reduce mortality, decrease antibiotic use, and improve the overall health of animals. OVT also has many other health beneficial properties, such as antioxidant, anti-inflammatory, anti-hypertensive, anticancer, and immuno-stimulating properties. These properties have been linked to intestinal health and animal performance metrics like body weight and daily weight gain. Thus, OVT plays a crucial role in animal health and performance and serves as a valuable agent in improving animal welfare.

## 1. Introduction

Chicken eggs carry an abundant number of proteins and minerals. The composition of a fresh raw egg consists of 76% water, and the remainder is protein, fat, ash, and carbohydrates. Indeed, the protein content is 10.9% in the egg white and 15.9% in the egg yolk [1]. Egg whites consist of multiple types of proteins that include lysozyme, defensins, ovostatin, cystatin, ovalbumin, and avidin, which are well known potent bacteriolytic proteins. Egg whites also include substantial quantities (12–13%) of ovotransferrin (OVT), promoting the growth and development of the chicken embryo, mainly by preventing the growth of microorganisms together with other proteins like lysozyme [2]. Antibiotic resistance poses a serious threat to livestock because of the overuse and misuse of antibiotics. If it rises continuously, 10 million annual antibiotic resistance-associated deaths are anticipated by 2050 from a variety of untreatable infections [3]. Therefore, the application of OVT in animal health and nutrition could provide benefits as a replacement of antibiotic use in helping control disease and illness. In this article, we attempt to describe the structure, stability, and beneficial effects of OVT towards the development of functional food ingredients and as important components of nutraceuticals for human and animal health.

## 2. Structure of OVT

**Figure 1:**
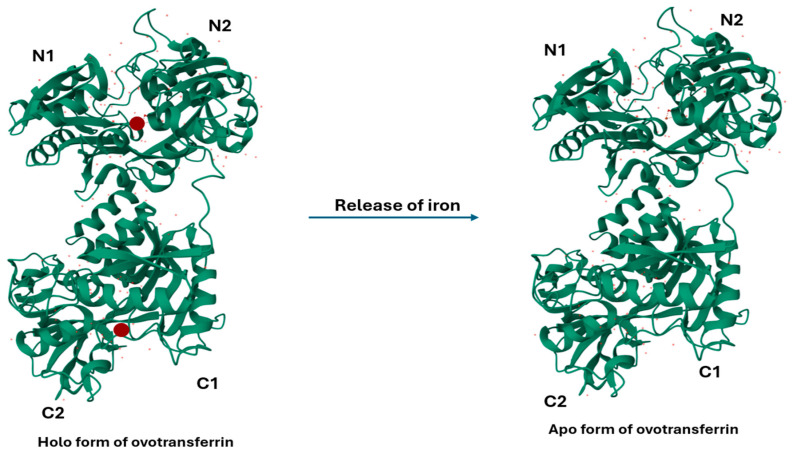
Refined crystallographic structure (at 2.4 angstroms resolution) of both holo- and apo- form of hen ovotransferrin with four domain C1/C2, and N1/N2. Holo-ovotransferrin is bound with diferric ion (red spheres) that release and convert into apo-ovotransferrin. The figure was produced and derived from RCSB (1OVT).

OVT, also known as conalbumin, is a monomeric metal-chelating glycoprotein belonging to the transferrin family. OVT is synthesized via the transcription of the transferrin gene in the oviduct and is then secreted at high levels in the egg white, which is regulated by progesterone and estrogen in the oviduct. OVT is composed of a single 686 amino acid polypeptide with a molecular mass of around 77.7 kDa [4]. Structurally, it has two globular lobes (N- and C-terminal) interconnected by an α-helix of nine amino acidic residues. As Figure 1 shows, each lobe consists of two α/β domains (N1 and N2, and C1 and C2, respectively), linked by two anti-parallel β-strands [5].

As OVT is a member of the transferrin family, each of its lobes has the capability to reversibly bind one Fe^3+^ ion along with one CO_3_^2−^ anion. Both lobes have different iron-binding properties despite both lobes displaying a high sequence homology. This difference is probably due to the presence of an extra interdomain disulfide bond (Cys478-Cys671) in the C-lobe, which weakens its affinity towards Fe^3+^ ions [6]. Iron-free (apo) and iron-bound (holo) OVT (Figure 1) participate in the precise regulation of iron uptake in the cells. The binding and release of iron is mediated through ferroportin and hepcidin molecules [7]. Additionally, OVT hydrolyzed with α-chymotrypsin and elastase has higher Fe^3+^-chelating activities than the native OVT [8]. Studies have shown that iron-conjugated OVT is more stable following physical, thermal, and chemical exposure compared to iron-free OVT. For example, a study with 200% iron saturation prevented the ethanol-induced denaturation of OVT [9]. OVT is also able to bind to other divalent cations like chromium, copper, manganese, zinc, nickel, cobalt, and cadmium with lower affinities compared to iron [10].

OVT has over 50% homology with bovine and human lactoferrin. OVT also has similar proteolytic activity to both human and bovine lactoferrin in catalyzing the hydrolysis of several synthetic substrates [11]. However, OVT has higher thermal stability at pH 7.0 and 9.0. Like OVT, lactoferrin has identical iron-binding sites with similar molecular and functional properties [12]. In an in vitro gastric barrier model, both 15% and 100% iron-saturated OVT were found to be better in terms of iron transport and barrier integrity [13]. Thus, OVT could be a potential substitute for lactoferrin, as OVT is cost-effective and readily available.

## 3. Stability and Bioavailability of OVT

OVT is the most heat-sensitive protein in egg white and is coagulated near 60 °C. The binding of iron to OVT (holo-form) makes it a salmon pink color and resistant to proteolytic hydrolysis and thermal denaturation, whereas a metal-free OVT (apo-form) is colorless and sensitive to physical and chemical treatments [14]. Compared to bovine lactoferrin, OVT has higher secondary structure stability at pH 7.0 and 9.0 with heating. Furthermore, the thermal aggregation degree of OVT was found to be lower than both bovine and human lactoferrin at pH 7.0 [12]. However, the stability of OVT can be further increased by its modification. A study showed that OVT-derived IRW (Ile-Arg-Trp) peptide is resistant to intestinal peptidase up to 60 min. In the study, OVT was found to be stable when IRW was transcellularly transported in Caco-2 cell monolayers; thus, the study suggested that absorbed IRW may remain intact at the site of action [15]. Additionally, OVT-derived peptide like IRW has been shown to be safe. It has been observed that feeding IRW for a period of 18 days does not affect body and organ weights, suggesting its safety in animals [16]. 

The thermal denaturation of proteins commonly occurs during food processing. Therefore, the thermal stability of functional proteins is required in order to retain their desirable biological activities. The ability to withstand thermal conditions is improved in two regards in OVT: (i) phosphorylation in the α-helix and β-sheet of OVT has been found to enhance the thermal tolerance of OVT by improving its structural orderliness and decreasing surface hydrophobicity [17] and (ii) dextran sulfate is also reported as a protein stabilizer and chaperone to OVT, enhancing its thermostability. Dextran sulfate has been shown to suppress the amorphous aggregation of OVT at pH 7.0 after heating. During heating, it is reported that dextran sulfate preserves nearly the entire secondary and tertiary structure of OVT through the strong electrostatic interactions of OVT with dextran sulfate, coupled with the reduced OVT hydrophobicity [18].

OVT has demonstrated bioavailability upon iron binding. Each lobe of OVT undergoes large conformational changes that cause the movement of lobes, which leads to bury metals, such as iron, inside the polypeptide chain. This conformational change improves its bioavailability [11]. Specifically, a holo-OVT 15 (152 ppm iron (15%) saturated form of OVT) showed high bioavailability compared to a free form of 100% holo-OVT. The experimental analysis of the basolateral environment of the gastric compartment of the GTL-16 cell showed slow but constant gastric absorption over time (1–3 h), confirming a “slow-release effect” of holo-OVT 15 [13]. Different evidence suggests that the absorption of OVT can be receptor-mediated transcytosis. Shirkhani et al. demonstrated that OVT can be taken up by IEC-6 cells as an intact protein from the apical surface and transported to the basolateral surface through trans-epithelial exocytosis [19].

## 4. Physiological Properties of OVT

**Figure 2:**
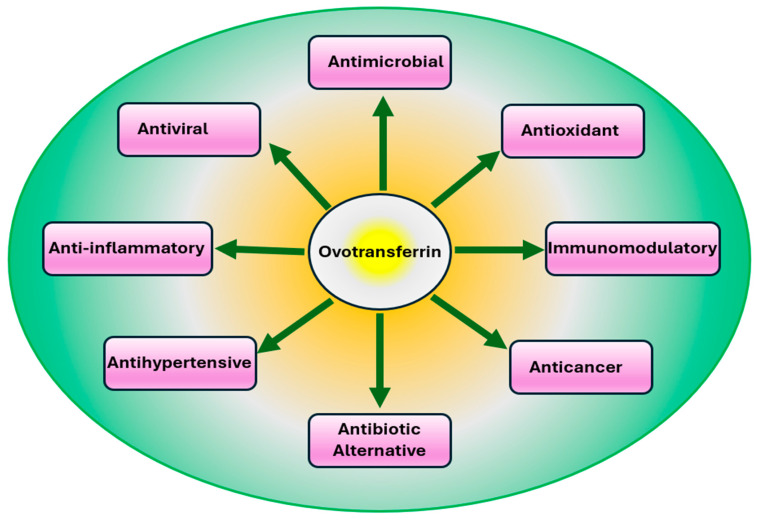
Physiological properties of ovotransferrin observed in both in vitro and in animal studies.

OVT is recognized as a major egg white functional protein with multiple bioactivities. OVT is known for iron binding, iron delivery, bacteriostatic, bactericidal, antiviral, antioxidant, anticancer, and immuno-stimulating properties (Figure 2). The derivatives and peptides produced from OVT are also reported to have antioxidant, antimicrobial, antihypertensive, and anticancer properties (Table 1). 

## 5. Antimicrobial Effects of OVT

**Figure 3:**
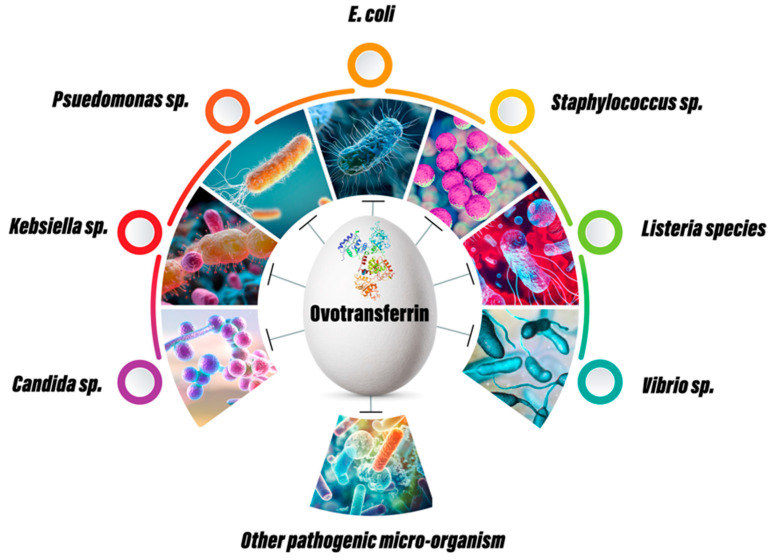
Antibacterial, antifungal and antiviral properties of ovotransferrin.

An antimicrobial agent is a substance that may be obtained from natural, synthetic, or semi-synthetic sources and has the property to stop the growth or kill microbes, particularly pathogenic ones. As a natural agent, OVT has shown tremendous antimicrobial effects in both in vitro and in vivo animal models (Figure 3).

### 5.1. In Vitro Studies of OVT

Eggs strongly resist microbial infection due to an arsenal of defensive systems that reside in the egg white and also, of course, due to the outer covering of eggshell. OVT is one of the major defensive proteins of egg white that has antimicrobial potency, including against Gram-positive and Gram-negative bacteria. As it has iron (Fe^3+^)-binding capacity, OVT serves as a potent bacteriostatic agent by starving the bacteria of iron and further hindering them from moving. OVT has additional antibacterial activity beyond iron chelation, which appears to depend on direct interactions with the bacterial cell surface, particularly the outer membrane, sequestering divalent ions from the outer membrane, and thus leading to membrane destabilization, membrane perturbation, and finally bacterial death [24]. Ibrahim et al. [20] have tested the antimicrobial efficacy of a cationic fragment of hen-derived OVT against *Escherichia coli*. This cationic fragment was found to cause the permeation of *E. coli* outer membranes via a self-promoted uptake that causes damage to the biological function of the cytoplasmic membrane, consequently killing Gram-negative bacteria.

The antimicrobial capability of OVT increases via the addition of certain compounds, other than iron, due to their interaction with the bacterial surface. For example, the addition of bicarbonate ions to OVT increases its antimicrobial activity, while citrate addition exhibits antagonistic effects to OVT’s antimicrobial function [46]. Another study also showed that a combination of ethylenediaminetetraacetate (EDTA) and lysozyme with OVT improved its antimicrobial activities. In the study, EDTA (2 mg/mL) plus OVT and NaHCO3 induced a reduction in *E. coli* cell growth. Lysozyme (1 mg/mL) plus OVT and NaHCO3 also resulted in a reduction in *E. coli* bacterial growth [47]. However, only EDTA was able to enhance the antibacterial activity of OVT against *Listeria monocytogenes* in commercial hams [9]. The antimicrobial effect of OVT in combination with nisin and some selected meat additives was also investigated against the growth of *L. monocytogenes*. OVT (40 mg/mL) strongly inhibited the growth of *L. monocytogenes* in brain heart infusion (BHI) broth but not in frankfurters (sausage). However, the combination of OVT (40 mg/mL) and nisin (1000 IU) inhibited the growth of *L. monocytogenes* in both BHI and frankfurters [48]. These studies suggest that the antimicrobial effects of OVT can be improved through combination treatment.

OVT has also shown bactericidal properties along with bacteriostatic. OVT induced the suppression of the *Bacillus cereus* bacteria, which are opportunistic, pathogenic, spore-forming microorganisms and are well known for spoilage events in the sector of pasteurized food products [22]. The inhibiting activity of OVT was also tested against different species belonging to the genus Candida. Among hundreds of strains, only *Candida krusei* exhibited a noticeable resistance to OVT, while the other species appeared to be sensitive [25]. The antibacterial activity of OVT against different bacterial species was studied in vitro. OVT was comparatively more sensitive to Pseudomonas sp., *E. coli*, and *Staphylococcus mutans* than *Staphylococcus aureus* and than Proteus and Klebsiella species [46].

*Chlamydia psittaci* infects a wide range of avian species, occasionally causing systemic illness in birds that results in substantial losses in the poultry industry. Considering this fact, OVT was tested against *C. psittaci* in Buffalo Green Monkey (BGM) kidney cells and HD11 chicken macrophages as artificial hosts. The pre-incubation of *C. psittaci* with OVT (0.5 to 5 mg/mL) prior to infecting BGM cells significantly lowered the infection rate. Interestingly, OVT was more effective than human and bovine lactoferrin in inhibiting bacterial irreversible attachment and cell entry [26].

Hen OVT protein was further examined for its antibacterial efficacy against disease-causing bacteria. In a study, OVT reduced the growth of Acute Hepatopancreatic Necrosis Disease (AHPND), therefore increasing the presence of *Vibrio parahaemolyticus* bacteria strains (M0904, TW01, and PV1) in shrimp. Thus, this finding positioned OVT as a promising agent against AHPND-causing bacteria [27]. A modified OVT mesoporous silica nanoparticle has also been developed to minimize the antibiotic resistance in pathogenic bacteria, which facilitates antibiotic delivery to the vicinity of the pathogenic bacteria. The OVT-modified nanoparticles effectively inhibited the growth of *E. coli* in both in vitro and in vivo *models* and exhibited potent antimicrobial properties [23].

### 5.2. In Vivo Studies

Controlling respiratory pathogens like *Chlamydophila psittaci* (formerly known as *Chlamydia psittaci*) in large-scale poultry production is challenging, often leading to respiratory diseases and high mortality. However, the daily aerosol administration of OVT (5 mg/animal) in turkeys for 12 days significantly reduced the respiratory disease incidence caused by *C. psittaci* infections and mitigated the occurrence of *Ornithobacterium rhinotracheale* and avian metapneumovirus. OVT-fed animals stayed healthy until the age of 9 weeks. Consequently, OVT reduced mortality by 46% while also lowering antibiotic costs [28]. Thus, OVT is effective not only in vitro but also in vivo as it suppresses microbial growth. Because the use of antibiotics is discouraged due to emerging antibiotic-resistant bacteria and the pollution of ecosystems, OVT could be a novel, sustainable alternative to the use of antibiotics.

## 6. Antiviral Properties of OVT

Besides the role of delivering iron to cells and inhibiting bacterial multiplication, OVT has also demonstrated antiviral activity towards chicken embryo fibroblast infection by Marek’s disease virus (MDV), an avian herpesvirus. Moreover, OVT was found to be more effective than human and bovine lactoferrins in inhibiting MDV infection, and interestingly, no correlation between antiviral efficacy and iron saturation was found [29]. The hen OVT fragments have also shown antiviral activity toward MDV infection in chicken embryo fibroblasts. These fragments, displaying sequence homology with two bovine lactoferrin fragments, exerted herpes simplex virus infectivity suppression in an in vitro model [49]. OVT also acts against vesicular stomatitis virus (VSV), which causes an acute, febrile vesicular disease in cattle, horses, and pigs. In a study with VSV-infected mouse peritoneal macrophage cells, OVT pretreatment suppressed the replication of VSV and increased the levels of type I interferon [30], indicating its potential against viral infection in animals.

## 7. Antioxidant Properties of OVT

Oxidative stress is the root cause of many diseases that result from an imbalance of free radicals and antioxidants. Free radicals, particularly reactive oxygen species (ROS) and reactive nitrogen species (RNS), oxidize cellular molecules like protein, fat, and genetic material, which leads to cell damage. However, antioxidants neutralize those free radicals and protect these cellular molecules from damage.

### 7.1. In Vitro Studies

OVT has been shown to be a superoxide dismutase (SOD) mimic protein with potent superoxide anion scavenging activity. OVT showed superoxide scavenging activity that was remarkably higher than the known antioxidant ascorbate. Interestingly, OVT has a unique specificity to scavenge superoxide but not oxidase inhibition. Moreover, metal-bound OVT has shown greater superoxide scavenging capacity than the metal-free OVT (apo-protein) [31], with the metal helping possibly in catalyzing the superoxide ion neutralization. Besides OVT, its derivatives IRW and IQW (a tripeptide) have been shown to attenuate tumor necrosis factor (TNF)-α-induced oxidative stress in endothelial cells by lowering TNF-induced superoxide generation [32]. Thus, OVT has remarkable superoxide scavenging activity and provides an opportunity for its use as an antioxidant agent.

Enzymatically hydrolyzed OVT has been reported to have improved functional activities compared to natural OVT. Hydrolysates of OVT, prepared by using promod 278P, thermolysin, and a combination of the two enzymes, showed strong antioxidant activities in the in vitro study. OVT exhibited antioxidant activity when analyzed using the DPPH assay, but OVT hydrolysates showed a strong free radical scavenging activity when both DPPH and NO- or ABTS-scavenging activity were measured [33]. Another study also confirmed that OVT hydrolysate has higher antioxidant activity than native OVT. OVT hydrolysate showed approximately 3.2 to 13.5 times higher superoxide anion scavenging activity than native OVT. However, both native and hydrolysate of OVT have protective effects against oxidative stress-induced DNA damage in human leukocytes [34]. Furthermore, hydrolysates obtained from the autoclaving of OVT have also shown better antioxidant activities in suppressing the discoloration of β-carotene effectively and in preventing the oxidation of linoleic acid [50]. Thus, it has been suggested that OVT has the potential to be used as a natural antioxidant in food.

The common tea component, catechin, is known for its excellent antioxidant activity. However, catechin antioxidant potency was found to be increased by the conjugation with OVT. Mechanistically, catechin covalently binds to lysine (residues 327) and glutamic acid (residues 186) in OVT, and this conjugate yielded higher oxygen radical scavenging activities, indicating that conjugation with catechin is an effective way to improve the antioxidant activity of the protein [51]. Besides this, OVT hydrolysate has also been shown to increase the antioxidant capacity of teas by adding either hydrolysates or its purified peptide IRW. However, OVT hydrolysate did not improve the antioxidant stability of teas [52]. This suggested that OVT hydrolysate could be used as a functional food ingredient in enhancing the antioxidant capacities of foods, which would benefit animal nutrition and further health.

### 7.2. In Vivo Studies

Besides being antimicrobial, OVT has demonstrated antioxidant activities in animals. In ethanol-induced gastric injury in mice, OVT improved gastric antioxidant ability by increasing SOD and glutathione levels and decreasing malondialdehyde and myeloperoxidase content. The antioxidant activity of OVT resulted in an improvement in gastric injury [35].

## 8. Anti-Inflammatory Properties of OVT

Inflammation is the body’s response to any harmful stimuli, including infection, injury, or irritants. If inflammation is acute, the body’s immune response fights off infection and heals damaged tissue. However, if inflammation persists, it may result in several inflammatory diseases. Anti-inflammatory molecules neutralize and protect the body from the harmful effects of inflammatory factors.

### 8.1. In Vitro Studies

OVT has displayed anti-inflammatory activities in various studies. In a study, OVT-derived tripeptide IRW has shown anti-inflammatory effects in human umbilical vein endothelial cells (HUVECs). When HUVECs were pretreated with IRW for 12 h before introducing lipopolysaccharide (LPS), IRW inhibited the LPS-induced enhancement of TNF-*α*, interleukin (IL)-8, intercellular cell adhesion molecule-1 (ICAM-1), and vascular cell adhesion molecule-1 (VCAM-1) expression in HUVECs [53]. Furthermore, IRW was found to be effective in LPS-neutralizing activity and inhibiting the LPS-induced activation of NF-κB and MAPK signaling pathways in Caco-2 cells. Along with IRW, another OVT-derived peptide, IQW, has also been shown to attenuate TNF-induced inflammatory responses in endothelial cells. Both IRW and IQW significantly inhibited the TNF-induced upregulation of ICAM-1, which was mediated by suppression of the NF-κB pathway [32], indicating its anti-inflammatory activities in experimental models.

### 8.2. In Vivo Studies

Besides in vitro models, OVT has also demonstrated anti-inflammatory activities in animals. In ethanol-induced gastric mucosal injury in BALB/c mice, OVT effectively downregulated the expression of inflammatory markers, such as TNF-α, IL-1β, and IL-6, but enhanced the secretion of IL-4, IL-10, and prostaglandin E2 [45]. Moreover, the pretreatment of OVT inhibited the activation of the MAPK/NF-κB pathway in mouse gastric mucosa [35]. In another mouse model of dextran sodium sulfate (DSS)-induced colitis, OVT has exhibited a potent anti-inflammatory effect. The feeding of OVT (50 or 250 mg/kg BW) for 14 days with DSS (to induce acute colitis) to mice resulted in reduced clinical signs, weight loss, shortening of the colon, and inflammatory cytokine markers of disease, suggesting it as a potential promising candidate for the prevention of inflammatory bowel diseases (IBD) [36]. In a continuation of the above findings, Chai et al. [37] showed that OVT-derived IQW peptide (60 μg/mL) alleviated not only DSS-induced colitis by enhancing the body’s anti-inflammatory ability but also regulated intestinal flora and metabolic changes. IQW (60 μg/mL) also restored weight loss, amended the liver index, and improved histomorphological and pathological changes in the colon compared to the DSS-treated group [37]. Additionally, IRW reduced the levels of TNF-*α*, IL-6, and myeloperoxidase (MPO) activity in the rats [53]. Thus, OVT and its peptide can combat inflammation both in vitro and in vivo.

## 9. Immunomodulatory Effects of OVT

The innate immune system of the body plays an important role in fighting against the causative factors of disease. However, the body also needs additional immunomodulators, which are the substances that change your body’s immune response, to effectively fight against pathogens and diseased cells like cancer.

### 9.1. In Vitro Studies

OVT has been shown to exhibit anti-inflammatory effects at low concentrations and immune-enhancing activity at high concentrations. For example, OVT (50 μg/mL) significantly inhibited the secretion and expression of inflammatory factors in LPS-stimulated RAW264.7 macrophages, without affecting cluster of differentiation (CD) 14 and toll-like receptor 4 (TLR4). However, OVT (200 μg/mL) alone not only enhanced the expression of the TLR4 gene but also increased the phagocytic activity and the production and expression of inflammatory factors [38]. OVT hydrolysate has been shown to induce immunity in bone marrow-derived dendritic cells (BMDCs). This hydrolysate induced dendritic cell (DC) maturation in terms of increasing the expression levels of histocompatibility complex class II (MHC-II) and the costimulatory molecules CD83 and CD86 and the production of TNF-α, IL-12p70, and RANTES. Furthermore, OVT hydrolysate improved the ability of LPS-stimulated DCs to induce allogeneic T lymphocyte activation [39]. An immune-enhancing effect was also shown by Lee et al. [40] using an in vitro model. They showed that OVT hydrolysates enhanced NO production by increasing iNOS expression in mouse macrophages. OVT hydrolysate treatment applied to RAW 264.7 macrophages also increased the production of pro-inflammatory cytokines and phagocytic activity, indicating OVT hydrolysates’ potential as immune enhancers [40].

An interesting report revealed that the OVT that was obtained from egg whites by hens immunized with bacterial antigens displayed immunological activity. This OVT could have potential in the prevention and treatment of infections resistant to antibiotics. This OVT has been shown to preserve immunological properties like immunoglobulin Y [54]. Thus, OVT promotes immunological factors and combats pathogenic infections.

### 9.2. In Vivo Studies

OVT has also shown immunomodulatory effects in animals. In a mouse model of cyclophosphamide-induced intestinal immunosuppression and injury, OVT has demonstrated intestinal immunomodulatory function by increasing the MHC-II and CD83 levels to enhance intestinal DCs maturation. Moreover, OVT promoted the expression of TNF-α, IFN-γ, IL-4, and IL-10. Furthermore, the imbalance ratio of Th1 and Th2 in the intestine was regulated to produce an immune response and regulate the expression of immunoglobulin A (IgA) and secretory IgA (sIgA), which indicates increased humoral immunity following OVT treatment in mice [41]. In another mouse model, OVT alleviated cyclophosphamide-induced immune dysfunction. OVT has been shown to improve the spleen and thymus indices and enhance the secretion of TNF-α, IL-10, and IgA, which indicates its immunomodulatory effects in animals [55]. As OVT and its hydrolysates stimulate immunity by enhancing dendritic cell maturation and IgA/sIgA production or modulating other immunomodulatory markers in small animals, they could also be effective in larger farm animals like pigs and cattle.

## 10. Others

OVT not only displays antimicrobial, antioxidant, and immunomodulating activities but can also suppress osteoclastogenesis. OVT inhibits osteoclast differentiation and bone resorption in mouse macrophage RAW 264.7 cells through the suppression of RANKL-induced NF-κB and MAPK signaling pathways. In addition, OVT induced the apoptosis of mature osteoclasts, accompanied by the increased gene expression of *Bim* and *Bad* and the decreased expression of *Bcl-2* and *Bcl-xl* [42]. Thus, egg white OVT acts as an inhibitor of osteoclastogenesis, which may be used for the prevention of osteoporosis.

OVT exhibits anticancer activity by suppressing the growth of various human cancer cells. However, OVT hydrolysates have exhibited stronger cytotoxic activities than the natural OVT in human cancer cell lines like gastric (AGS), intestine (LoVo), colon (HT-29), and cervical (HeLa) cancer cells [33]. Another study on OVT and its hydrolysate showed that they can induce cytotoxicity in various other cancer cells, including the lung (A549 and SK-MES-1), breast (MCF-7), larynx (Hep-2), and liver (HepG2). Both showed remarkable effects on inducing toxicity; however, OVT hydrolysate has a more potent effect than OVT [56]. The fact that the exposure of OVT hydrolysates to AGS, LoVo, HT-29, and HeLa cancer cells displayed the strongest cytotoxic activity compared to OVT is further confirmed by Lee et al. OVT hydrolysate has also been shown to inhibit cell proliferation and induce apoptosis in human colon cancer (HCT-116) and breast cancer (MCF-7) cells without affecting normal human mammary epithelial cells (HMECs), indicating its specificity towards cancer cells. This anticancer effect in colon and breast cancer was found to be associated with the collapse of mitochondrial membrane potential and caspase-9 and -6 activation, indicating the involvement of the mitochondrial pathway in cytotoxicity [43].

In addition to anticancer activity, OVT hydrolysate (by the promod 278P enzyme) displays angiotensin-converting enzyme (ACE)-inhibitory activities. ACE participates in narrowing blood vessels, which can lead to high blood pressure and ultimately heart disease. OVT has been shown to display strong ACE-inhibiting activity, with an IC50 value of 1.53 ± 0.20 mg/mL. However, at 10 mg/mL level, the hydrolysate of OVT showed a 77% inhibition of ACE-inhibitory activity [44]. This result indicated that the hydrolysate of OVT has great potential as an antihypertension agent.

OVT-derived IRW peptide also inhibits the ACE, resulting in a reduction in blood pressure in spontaneously hypertensive (SH) rats through reduced vascular inflammation and increased nitric oxide-mediated vasorelaxation. An IRW (3 and 15 mg/kg) treatment applied to these rats attenuated the mean blood pressure by ~10 mmHg and ~40 mmHg at the low- and high-dose groups, respectively, compared to untreated SH rats [44]. Furthermore, Majumder et al. [16] found that it contributes to antihypertensive activity through increased ACE2 and decreased pro-inflammatory gene expression. Lee et al. [57] identified another ACE inhibitory peptide derived from hen OVT. This peptide showed a concentration-dependent inhibition of ACE activity in vitro with an IC50 value of 102.8 μM. Moreover, the intravenous administration of this peptide and its hydrolyzed product into SH rats produced the maximal reduction in systolic blood pressure at 40 min and 20 min after injection, respectively [57]. Thus, these findings suggest that OVT and its hydrolyzed products display antihypertensive effects through the inhibition of ACE activity.

Acute gastric mucosal injury is a common gastrointestinal disorder. OVT is reported to reduce gastric disease caused by *Helicobacter pylori* infection in the intestine of immunosuppressed mice. Furthermore, OVT has been shown to be effective reducing ethanol-induced gastric mucosal injury in BALB/c mice [45]. The findings of this study further indicate that OVT could be an important protein against gastric injury caused by *H. pylori* infection.

## 11. Conclusions

A balanced diet is important for all livestock animals, providing the necessary nutrients to grow, develop, reproduce, and provide strong immunity against infections. In addition, animals need well-formulated diets to meet their needs at different life stages of growth and development. Deficiencies of balanced diets severely impact the growth, development, and production of animals. In prolonged deficiencies, animals will experience various diseases, disorders, or even fatalities. However, in recent years, nutritional research has contributed to the development of nutritional feed additives for the improvement of animal health and growth and reducing incidences of animal illness [58].

**Figure 4:**
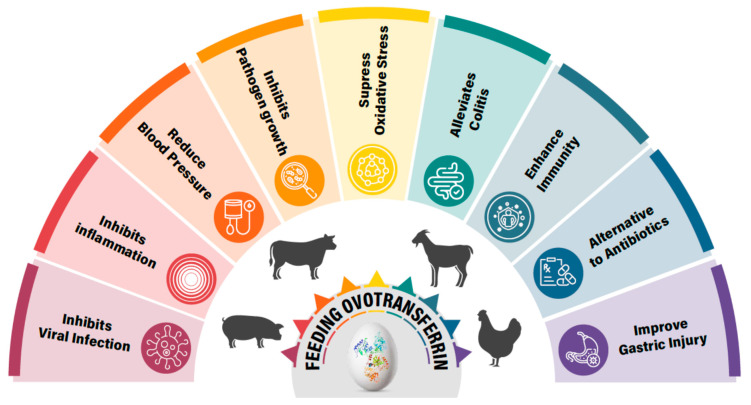
Ovotransferrin feeding appears to provide overall health benefits to animals as it displays multiple physiological properties.

OVT, which has demonstrated various physiological properties, including antimicrobial, antioxidant, antiviral, and immunomodulatory properties, could be an important nutritional feed additive for animals. OVT has shown efficacy against various pathogenic bacteria and viruses, as well as inducing immune responses and suppressed oxidative stress in animals. Thus, these properties of OVT show promise in further improving animal health and production (Figure 4). Interestingly, OVT-hydrolyzed peptide has shown better efficacy than natural OVT in reducing oxidative stress and inflammation, which opens a new avenue for further research and use. OVT has emerged as a potential ingredient for animal feed due to its multiple health benefits. However, the regulatory landscape and market potential for OVT in animal feed are complex. Therefore, regulatory hurdles and market challenges need to be addressed, if any, before use in animal feed. So far, most of the studies have been conducted small scales. Therefore, to determine its beneficial effects, large-scale feeding trials are required, which will also help in exploring the pharmacokinetic behavior and the synergistic effects with probiotics and prebiotics.

## Figures and Tables

**Table 1:** In vitro and in vivo physiological properties of OVT.

Effects of OVT	Model	Dose	Reference
Anti-microbial properties			
Killed bacteria by permeation	*E. coli*	2 μM	[20]
Permeation of K^+^ ion in the bacterial cell membrane	*E coli*	13 μM	[21]
Provoked perturbation of the electrochemical potential of the cytoplasmic membrane	Bacillus cereus group	13 g/L	[22]
OVT-derived nanoparticles interacted with bacteria	*E. coli*	20 mg/kg	[23]
Induced outer membrane permeabilization	Salmonella enteritidis	13 g/L	[24]
Interacted with fungal surface proteins and inhibited growth	Candida albicans and CC. krusei	1 mg/mL	[25]
Inhibited infectivity, adhesion, and invasion in a dose-dependent manner	*Chlamydophila* Psittaci	0.5–5 mg/mL	[26]
Prolonged the lag phase of bacteria	Vibrio parahaemolyticus	1–10 mg/mL	[27]
Reduced respiratory disease as aerosol	Chlamydia psittaci	5 mg/animal	[28]
Antiviral properties			
Inhibited viral antigen synthesis	HSV-1 and MDV	<3 mg/mL	[29]
Downregulated RNF125 and upregulated RIG-I expression by NF-κB; results increased IFN-1 expression in macrophages	VSV	100 ng/ml	[30]
Antioxidant properties			
Acted as SOD mimic protein and scavenged superoxide anion (O^2−^)	Xanthine/xanthine oxidase coupling system	6.4 μM	[31]
OVT-derived peptides reduced TNF-induced superoxide generation	Endothelial cells	50 μmol/L	[32]
OVT hydrolysates showed strong free radical scavenging activity	Radical scavenging assay	0.5–2 mg/mL	[33]
OVT and OH-OVT protected against oxidative stress-induced DNA damage	Human leukocytes	500 μg/mL	[34]
Anti-inflammatory effects			
Inhibited MAPK/NF-κB Pathway	Gastric epithelial cells	50−400 μg/mL	[35]
Prevented DSS-induced colitis by inhibiting epithelial dysfunction and modulating cytokine profile	BALB/c mice	50 or 250 mg/kg BW/day)	[36]
OVT-derived peptide (IQW) mitigated disulfide sodium-induced colitis	C57BL/6J mice	20–100 μg/mL of IQW	[37]
Immunomodulatory effects			
Regulated TLR4-mediated NF-κB/MAPK signaling	RAW 264.7 Ms. macrophage cells	50 μg/mL	[38]
OVT-hydrolysates inhibited DC maturation by reducing MHC-II, TNF-α, IL-12p70, and RANTES	Bone marrow-derived dendritic cells	250 μg/mL	[39]
OVT hydrolysate promoted phagocytic activity by inducing NO and iNOS	RAW 264.7 macrophages	250–500 μg/mL	[40]
Promoted maturation of intestinal DC and increased cytokine expression	Kunming mouse	2–200 mg/kg	[41]
Other properties			
Induced apoptosis of mature osteoclasts by increasing expression of Bim and Bad	Mouse osteoclast	1–1000 μg/mL	[42]
OVT hydrolysates exhibited cytotoxic properties against cancer cells	Human cancer cell lines	10 mg/mL	[33]
Autocleaved OVT dissipated mitochondrial membrane potential and activation of caspase-9/-6	HCT-116 and MCF-7	250 μg/mL	[43]
OVT hydrolysates acted as antihypertension agents by suppressing ACE	Human cancer cell lines	IC50: 1.53 ± 0.20 mg/mL	[44]
OVT-derived IRW peptide inhibited blood pressure through ACE reduction	Spontaneously hypertensive rats	3 and 15 mg/kg	[44]
Reduced gastric disease caused by *Helicobacter pylori*	BALB/c mice	50−400 μg/mL	[45]

OVT, ovotransferrin; RNF125, ring finger protein 125; RIG-1, retinoic acid-inducible gene I; NF-κB; SOD, superoxide dismutase; IFN-1, interferon 1; OH-OVT, hydrolysate ovotransferrin; NO, nitric oxide; iNOS, inducible nitric oxide synthase; TNF, tumor necrosis factor; MAPK, mitogen-activated protein kinase; TLR4, toll-like receptor 4, MHC-II, major histocompatibility complex; IL, interleukin; DC, dendritic cells; ACE, angiotensin-converting enzyme; HSV, herpes simplex virus; VSV, vesicular stomatitis virus; MDV, Marek’s disease virus.

## Data Availability

No new data were created or analyzed in this study. Data sharing is not applicable to this article.
